# Breastfeeding Behavior in Brazilian Children with Congenital Zika Syndrome

**DOI:** 10.1155/2020/1078250

**Published:** 2020-03-16

**Authors:** Alidianne Fábia Cabral Cavalcanti, Yêska Paola Costa Aguiar, Adriana Suely De Oliveira Melo, Alessandro Leite Cavalcanti, Sergio D'Ávila

**Affiliations:** ^1^Assistant Professor, School of Dentistry, State University of Paraiba, Campina Grande, Brazil; ^2^School of Dentistry, State University of Paraiba, Araruna, Brazil; ^3^Professor, School of Medicine, Federal University of Campina Grande, Campina Grande, Brazil; ^4^Associate Professor, Postgraduate Program in Dentistry, State University of Paraiba, Campina Grande, Brazil

## Abstract

Congenital Zika Syndrome (CZS) is a condition that has emerged only recently, bringing together multiple changes, including significant changes in the stomatognathic system, which may compromise sucking behavior and consequently the breastfeeding practice. The aim of this study was to investigate the breastfeeding behaviors in children with CZS. A longitudinal study was carried out in two reference centers in Northeastern Brazil. The nonprobabilistic sample consisted of 79 children diagnosed with physical, neurological, and behavioral alterations compatible with CZS. Information regarding the child, nutritive, and nonnutritive sucking behavior and changes related to the sucking reflex was collected. Data were presented through descriptive and inferential statistics. In the bivariate analyses, the chi-squared test was used and 5% significance level was adopted. The majority of children had severe microcephaly (59.7%). Breastfeeding was performed at birth in most of CZS children (89.9%) but only 36.6% of them presented exclusive breastfeeding in the six months of life. Bottle feeding and pacifier were used in 89.9% and 55.7%, respectively. Sucking and swallowing difficulties and occurrence of gastroesophageal reflux were observed in 27.8%, 48.0%, and 29.2% of children, respectively. Early weaning was associated with bottle feeding (*p*=0.005) and pacifier sucking (*p*=0.003). Although breastfeeding practice at birth constitutes a behavior adopted by most of mothers, adherence to this exclusive habit until the first six months of life was low since the children presenting a large number of comorbidities with direct interference in the suction reflex, sucking, and swallowing difficulty.

## 1. Introduction

Breastfeeding has multiple benefits in the short or long term, providing advantages for both infant and mother. There are reports about the effects of breastfeeding on the decline of infection rates and positive impact on physical, cerebral, and cognitive development of infants [1–4].

The World Health Organization [5], the United Nations Children's Fund [6], and the Brazilian Ministry of Health [7] advocate exclusive breastfeeding until six months of age and complementary feeding until at least the second year of life. However, even considering this strong recommendation, it is estimated that in low- and middle-income countries, only 37% of children younger than 6 months are exclusively breastfed; an even lower percentage is observed in high-income countries [8].

In Brazil, the estimated average time of exclusive breastfeeding for children younger than 6 months was 41%, and significant differences were identified among the five Brazilian macro-regions, and even among the states that compose the same region. In northeastern Brazil, most of federal units, including the state where the study was conducted, showed breastfeeding prevalence lower than the national average [9].

In the context of zika virus transmission, recommendation similar to that directed to other newborns, i.e., infants born from mothers with suspected, probable, or confirmed zika virus infection, was published, advocating breastfeeding within 1 hour after birth, exclusive breastfeeding for 6 months, introduction of complementary foods, and continue breastfeeding up to 2 years of age [10].

The alert was released after the unexpected growth of live births with microcephaly in 2015, which occurred after the occurrence of fever due to the zika virus. The hypothesis of causality was confirmed and in addition, it was verified that the implications in children extrapolated the occipitofrontal reduction of the head, and a set of alterations denominated congenital zika syndrome was established [11, 12].

More recently, changes in lip tonus have also been reported, with direct repercussions on sucking and subsequent sealing during breastfeeding in children with congenital zika syndrome (CZS) [13]. In addition, a marked oral dysfunction has been described, with dystonic movements of the tongue, lack of pharyngeal sensitivity, increased risk of aspiration, and episodes of severe dysphagia [14, 15].

Considering the above and that congenital zika syndrome is a new condition and, to date, there are no studies that present the prevalence of nutritional sucking habits of this population, the aim of this article was to investigate the breastfeeding behaviors in Brazilian children with congenital zika syndrome. Assuming the premise that CZS causes changes in the stomatognathic system, the hypothesis adopted is that children affected by this condition will present changes in the exclusive breastfeeding pattern.

## 2. Materials and Methods

### 2.1. Study Design

This is an observational, longitudinal, descriptive-analytical study with quantitative approach.

### 2.2. Study Location

The research was developed in two rehabilitation centers that offer assistance to children with congenital zika syndrome, providing physical, intellectual, auditory, and visual rehabilitation treatment.

### 2.3. Population and Sample

The study population comprised a total of 98 children with clinical and neurological changes suggestive of congenital infection by the zika virus. The nonprobabilistic sample included 79 children with confirmed diagnosis of CZS and the youngest child included was two months old and the oldest child was 17 months old. The children were followed from September 2016 to March 2018. Nineteen excluded children had neonatal hypoxia and syphilis-related congenital infection as etiological agent.

### 2.4. Data Collection

Data were collected through a face-to-face interview with mothers in the referred centers by two researchers and recorded in a specific form. This period was necessary to assess variables such as sucking difficulty and swallowing disturbance and the presence of gastroesophageal reflux. The analyzed variables included information regarding the child such as sex, microcephaly occurrence (-2 standard deviations below the mean for sex and gestational age, with -3 standard deviations below the mean representing severe subtype) [16], nutritive suction behavior (breastfeeding at birth, exclusive breastfeeding (until six months of age), early weaning (interruption of breastfeeding before the child's six months of life), and use of bottle for cow's milk or artificial milk, nonnutritive sucking behavior (pacifier and finger sucking), comorbidities with direct interference in the sucking reflex (sucking and swallowing difficulties), and presence of gastroesophageal reflux.

Only infants breastfed or fed with express breast milk, without artificial milk supplements, formulas, or solid foods, were considered to be exclusively breastfed. The use of vitamin supplements and medications was accepted [17].

### 2.5. Statistical Analysis

Data were entered in a database in the Statistical Package for Social Sciences, Version 21.0 (IBM Corporation, Chicago, IL, USA) and presented through descriptive statistics (absolute and percentage distributions and total amplitude-minimum and maximum values). The chi-squared test was used to investigate possible associations between presence of “early weaning” and variables “bottle feeding” and “pacifier use”. The significance level was set at 5%.

### 2.6. Ethical Aspects

The study was evaluated and approved by the Research Ethics Committee of a public university, under the terms of protocol No. 2.040.765, which is carried out in accordance with the principles contained in the Declaration of Helsinki and in accordance with Brazilian provisions. All mothers were informed about the aims and procedures of the study and signed the informed consent form.

## 3. Results

The distribution of children according to sex was similar (50.6% of girls and 49.4% of boys). High percentage (79.5%) of infants exhibited reduced the head circumference at birth, with severe microcephaly occurring in 59.7% of patients (Table 1).

Analysis of sucking habits revealed that 89.9% of children had been breastfed at birth; however, an expressive percentage had the habit interrupted before the age of six months (63.4%). Regarding breastfeeding duration, variation from 1 month to 29 months was also verified. The use of bottle was reported by 89.9% of mothers, while nonnutritive sucking, represented by pacifier use, was present in 55.7% of children and digital sucking was practiced by only 3.8% of the sample. Regarding comorbidities with direct interference in the suction reflex, sucking and swallowing difficulty was reported by 27.8% and 48.0% of mothers, respectively. The presence of gastroesophageal reflux affected 29.2% of children (Table 2).

## 4. Discussion

Despite the importance for the child's development, breastfeeding practice is declining throughout the world [18]. In addition, there are reports of discontinuation of breast milk supply as the child grows. This reality is present in the routine of typical [19] or atypical infants (syndromic) [17, 20].

In this investigation, it was possible to observe high frequency of mothers who, immediately after giving birth, started breastfeeding. However, six months after, the number of mothers who interrupted breast milk supply to infants was expressive. A previous study has shown that 47.1% of children with microcephaly associated with congenital zika virus infection were exclusively breastfed [21]; a result higher than 36.6% identified in the present investigation. Therefore, the hypothesis adopted in this study that children affected by this condition will present changes in the exclusive breastfeeding pattern was confirmed. The prevalence of breastfeeding in nonsyndromic children ranges from 62% in Bangladesh [22] to 79% in Hawaii [23].

Recent studies carried out with a population of children with Down Syndrome have described that the reasons reported by mothers to justify exclusive breastfeeding suspension include intercurrences with children such as hypotonia that significantly affected suction, drowsiness, low weight gain, and recurrent hospitalizations [17, 20].

In the specific case of CZS, in addition to hypotonia and suction disorders [13], prolonged feeding and occurrence of dysphagia were reported in different levels of severity [15]. It is noteworthy that the dysphagia outcome, present in cases of congenital microcephaly caused by the zika virus, may develop in children older than 3 months of age, and, as a rule, phenotype shows greater severity, including children with not very serious neurological manifestations [15, 24].

Despite the unavailability of information on the videofluoroscopic findings in these children, at the time of data collection, the gold standard method for inference of the medical diagnosis of dysphagia and/or aspiration [15], the hypothesis that early weaning may have been influenced by the existence of swallowing disorders is robust due to the current findings that revealed high prevalence of this alteration in the children evaluated. Other authors have also reported the occurrence of coughs, regurgitations, reflux, and choking [13, 15].

Regarding the clinical signs indicative of dysphagia, in addition to speech therapy, the use of thickeners was incorporated into the children's diet as a strategy adopted in an attempt to improve the oral sensory-motor skills of children and, consequently, the swallowing process. However, in situations in which no success using thickeners was achieved and there were persistent episodes of choking, the nasogastric tube was installed. In a small group with considerable weight loss and establishment of malnutrition, gastrotomy and placement of the catheter through the abdominal wall were performed. According to these findings, it was verified that treatment of pediatric dysphagia should be individualized, taking into account the different stages of child development and the fact that for some children, there has been previous experience of oral feeding [25].

Feeding problems in children with neurological diseases are mainly explained by the existence of brain damage that lead to poor swallowing coordination, posture abnormalities, and digestive tract motility, such as gastroparesis and gastroesophageal reflux [24, 26].

Although these problems may be the cause of dysphagia observed in infants with CZS, CZS-associated dysphagia may also be caused by abnormalities of the orofacial anatomy, sensitivity of the upper and oral respiratory tract, and changes in upper gastrointestinal tract function caused mainly or secondarily by the direct action of the virus [15].

It is also worth pointing out that the use of pacifiers and bottles has also been considered a strong risk factor for early weaning [27–30]. The biological plausibility of this association is based on the dysfunction of muscle, lip, and tongue dynamics caused by the use of these devices [31], leading to inappropriate sucking behaviors [32].

The use of the bottle showed variability in relation to the children's age, with some of them using it from birth, while for others it was introduced in the first months of life. As reported by mothers, for many children, bottle was the device that provided food, especially in situations with the use of thickeners due to limitations imposed by dysphagia. Therefore, due to sucking difficulty and swallowing alterations, mothers fed their children compressing the bottle, so that the portion to be swallowed slowly and continuously dripped into the child's oral cavity.

With regard to the use of pacifiers, it is known that this is a widespread cultural habit among Brazilian children [30, 33], and therefore, their use was not different in the investigated population, given the high percentage found. The pacifier's offer was mainly motivated by the need to comfort and calm the child, similar to what occurs with typical children, since constant and difficult control crying is present in the first months of life. The extreme irritability of these children is possibly associated with muscle spasms and/or epilepsy, hyperexcitability to external stimuli [15], or as they develop, it may be one of the many signs and symptoms present in the phase of deciduous teeth eruption [34].

The prevalence of digital suction was low and may be due to the set of musculoskeletal alterations in most of the children under study. Thus, the hypothesis is that the congenital contractures in the upper limbs and consequent limitation of movements of wrists and hand fingers made children commonly present clenched fists, preventing them from carrying the hand and especially fingers to the mouth, reducing the practice of digital sucking.

The literature shows that nonnutritive sucking habits, when persistent, may be associated with greater chances of establishing anterior open bite. This change in dental occlusion can also be observed in children who were not breastfed [35]. Therefore, the prolonged breastfeeding practice seems to have very positive effects, not only by preventing the acquisition of nonnutritive sucking habits but also by directly stimulating the proper development of dental arches [33, 35].

It is imperative to develop prospective cohorts with the aim of evaluating the effect of these habits as well as of other possible risk factors for the establishment of dental malocclusion in children with CZS.

In view of the above, this pioneering study on the theme involving the breastfeeding pattern in children with CZS brings important findings that will enable a better understanding of the potential problems that can affect the stomatognathic system, both by physicians and dentists, as well as by other health professionals involved, such as speech therapists and physiotherapists, among others. The regular and continuous monitoring of these children in the different stages of child development is necessary and essential in order to provide better health conditions, mitigating the serious implications caused by the congenital zika syndrome.

## 5. Conclusion

For most children with congenital zika syndrome, breastfeeding was established in the first hours of life, but discontinuation of breast milk supply resulted in low frequency of exclusive breastfeeding in the first six months of life. The children present a large number of comorbidities with direct interference in the suction reflex, sucking, and swallowing difficulty.

## Figures and Tables

**Table 1:** Characterization of infants according to sex, presence of microcephaly at birth, and severity.

Variables	*N*	%
Sex of child		
Male	39	49.4
Female	40	50.6

Presence of microcephaly at birth		
Yes	62	79.5
No	16	20.5

Severe microcephaly		
Yes	37	59.7
No	25	40.3

**Table 2:** Distribution of children according to the breastfeeding pattern, nonnutritive sucking habits, and presence of gastroesophageal reflux.

Variables	*N*	%
Breastfeeding at birth		
Yes	71	89.9
No	8	10.1

Exclusive breastfeeding in the six months of life		
Yes	26	36.6
No	45	63.4

Early weaning		
Yes	45	63.4
No	26	36.6

Bottle use		
Yes	71	89.9
No	8	10.1

Pacifier sucking		
Yes	44	55.7
No	35	44.3

Digital suction		
Yes	3	3.8
No	76	96.2

Sucking difficulty		
Yes	22	27.8
No	57	72.2

Swallowing disturbance		
Yes	36	48.0
No	39	52.0

Gastroesophageal reflux		
Yes	21	29.2
No	51	70.8

Early weaning was associated with bottle feeding (*p*=0.005) and pacifier sucking (*p*=0.003) (Table 3).

**Table 3:** Association between occurrence of early weaning and use of bottle and pacifier sucking.

Variables	Early weaning				*p* value
	Yes		No		
	*n*	%	*n*	%	
Bottle use					
Yes	44	68.8	20	31.2	0.005^(1)^
No	1	14.3	6	85.7	

Pacifier sucking					
Yes	32	78.0	9	22.0	0.003^(2)^
No	13	43.3	17	57.7	

^(1)^Fisher's exact test; ^(2)^chi-squared test.

## Data Availability

The frequency and percentage data used to support the findings of this study are included within the article.
